# Vasocutaneous fistula formation and repair following inguinal hernia repair in a rhesus monkey (*Macaca mulatta*)

**DOI:** 10.1111/jmp.12569

**Published:** 2022-02-07

**Authors:** Parviz K. Kavoussi, Gregory Wilkerson, Stanton B. Gray

**Affiliations:** ^1^ Austin Fertility & Reproductive Medicine/Westlake IVF Austin Texas USA; ^2^ Keeling Center for Comparative Medicine and Research MD Anderson Cancer Center University of Texas Bastrop Texas USA

**Keywords:** *Macaca mulatta*, rhesus monkey, vasocutaneous fistula

## Abstract

A 6‐year‐old adult male rhesus macaque (*Macaca mulatta*) developed a vasocutaneous fistula following an anatomic inguinal hernia repair years earlier. The vasocutaneous fistula was surgically repaired, the vas deferens was ligated, and the wound was closed in layers with non‐overlapping suture lines with no further adverse sequalae of events.

## 1 INTRODUCTION

An inguinal hernia is a defect in the myofascial plane of the oblique and transversalis muscles and fascia allowing for herniation of intraabdominal or extraperitoneal organs through the defect.^1^ Human inguinal hernia patients have higher proportions of type III collagen than type I collagen; type III collagen has a lesser tensile strength than type I.^2^ Over 20 million inguinal hernia repairs are performed worldwide annually in humans.^3^ Inguinal hernias may be treated with anatomic surgical repairs or more commonly with mesh repairs. Surgical complications reported in humans include seroma, hematoma, urinary retention, hernia recurrence, chronic pain, and surgical site infection. Elective hernia repair has an overall complication rate of approximately 10%.^2^

Non‐human primates (NHPs) also experience inguinal hernias, but there is a paucity of data on incidence and complications. NHPs generally, and rhesus macaques specifically, are important animal models for various applications in biomedical research owing to their close phylogenetic relationship and anatomic and physiologic similarities to humans.^4^, ^5^

This report describes the clinical presentation, biopsy and histopathology, treatment, and surgical repair of a vasocutaneous fistula in a 6‐year‐old adult male rhesus macaque (*Macaca mulatta*), which developed as a complication of a non‐mesh anatomic inguinal hernia repair performed 5 years previously when the animal was a juvenile. A vasocutaneous fistula is an abnormal connection formed between the vas deferens and the skin. This is the first report to our knowledge of a vasocutaneous fistula developing as a complication of an inguinal hernia repair in humans or NHPs.

## 2 MATERIALS AND METHODS

The rhesus macaque described in this case report was housed and maintained at The University of Texas MD Anderson Cancer Center (UTMDACC), Michael E. Keeling Center for Comparative Medicine and Research (KCCMR) in Bastrop, TX. The animal was part of the closed breeding colony of Indian‐origin rhesus macaques (*Macaca mulatta*) at the KCCMR, which is specific pathogen free (SPF) for Macacine herpesvirus‐1 (Herpes B), Simian retroviruses (SRV‐1, SRV‐2, SIV, and STLV‐1), and *Mycobacterium tuberculosis* complex. All animal clinical care and manipulations were approved by the Institutional Animal Care and Use Committee (IACUC) and was in accordance with institutional guidelines (IACUC protocol #0804‐RN03). Animal care and husbandry conformed to practices established by the Association for the Assessment and Accreditation of Laboratory Animal Care (AAALAC), The Guide for the Care and Use of Laboratory Animals, and the Animal Welfare Act. All animals were socially housed in shaded, temperature‐regulated indoor–outdoor enclosures with numerous barrels, perches, swings, and various feeding puzzles, and substrates to mimic natural foraging and feeding behaviors. Standard monkey chow, *ad libitum* water, and novel food enrichment items were provided daily.

The KCCMR is located in a rural area on 381 acres with numerous trees around the perimeter of the colony. The SPF rhesus macaque breeding colony was founded in 1975, and has been a closed colony since approximately 1985. The UTMDACC KCCMR has been continuously AAALAC accredited since 1979.

## 3 RESULTS

**FIGURE 1:**
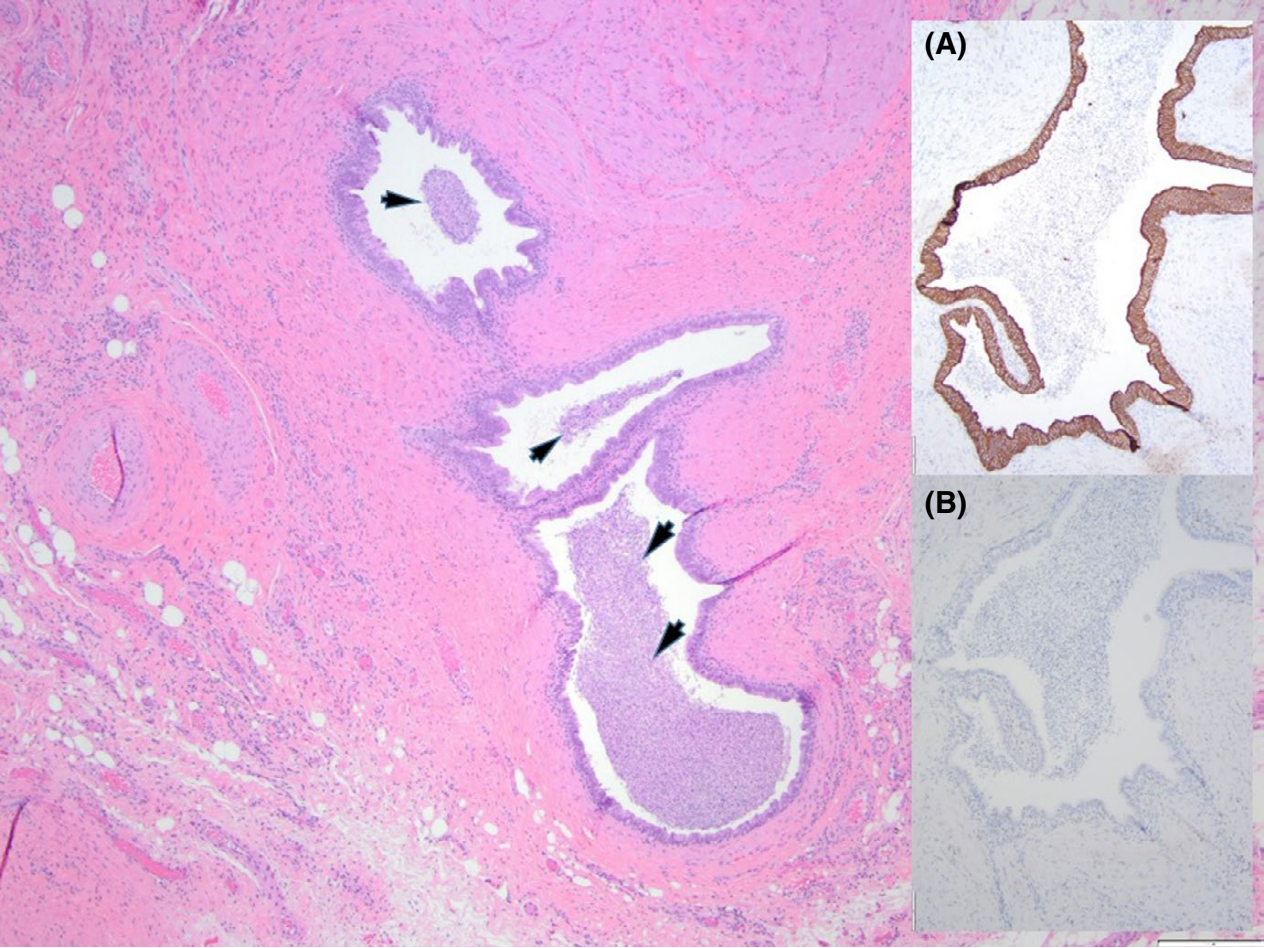
Multiple profiles of vas deferens containing luminal spermatozoa (arrowheads) are identified within the deep subcutaneous tissues. H&E stain at 40× magnification. Inset A: Slide stained with AE1/AE3 primary antibody for cytokeratin (epithelial cell) staining, 200× magnification. Inset B: Slide stained with CD68 primary antibody for tissue macrophage staining, 200× magnification

**FIGURE 2:**
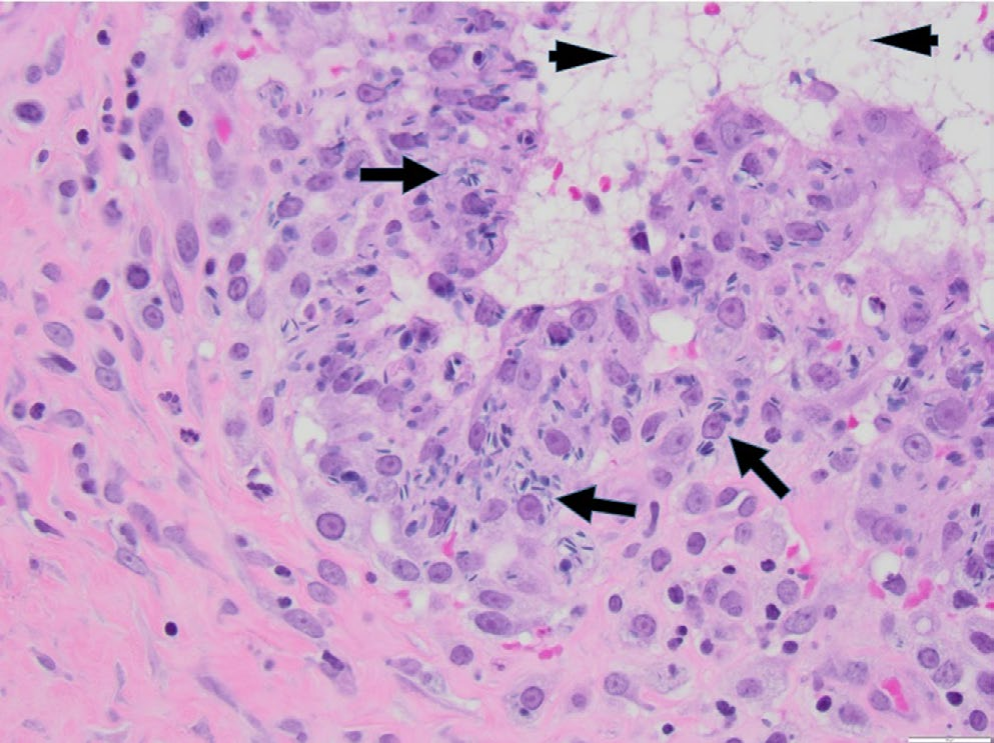
Fistulous tracts are lined with spermatozoa‐laden macrophages (arrows) and often contained proteinaceous to fibrinous fluid (arrowheads). H&E stain at 400× magnification

The 6‐year‐old male rhesus macaque in this case report underwent left inguinal hernia repair at approximately 1 year of age by an off‐site veterinary surgeon. An anatomic repair without mesh was performed. Approximately 5 years later, the animal presented with an approximately 1.5‐cm‐diameter red nodule in the left inguinal region at the site of the hernia scar. The small red nodule had been noted to wax and wane culminating in a small amount of clear drainage and then apparently resolve when in his harem breeding group. There were no signs of pain or discomfort, or anything abnormal noted in his behavior. Differential diagnoses included: encapsulated foreign body (e.g., suture) from prior hernia repair surgery, trauma, insect bite, and cutaneous hemangioma. Complete blood count and serum chemistry were unremarkable (mild elevation in creatinine kinase 2817 IU/L and mild monocytosis 0.81 × 10^3^/µl). This was initially managed by the onsite veterinarian by cleaning the red draining nodule with a dilute iodine solution and administering oral penicillin, but the draining sinus recurred approximately 6 weeks later. A culture of the draining tract was not performed. The animal continued to exhibit normal behavioral patterns within his social group. The on‐site veterinarian performed a cutaneous biopsy of the draining sinus in an elliptical pattern with approximately 1 cm margins, noting that the tract appeared to extend into the dermis. The animal was treated with injectable Penicillin G Benzathine (50 000 U/kg every other day for 6 days) and oral carprofen (2.2 mg/kg twice a day for 3 days). The animal was anesthetized with ketamine hydrochloride at 10 mg/kg for both exams and biopsy. The onsite veterinary pathologist assessed histopathology of the biopsy which revealed vas deferens with spermatozoa (Figure 1). The pathology specimen grossly revealed a mottled red‐tan tissue specimen measuring 2.0 × 1.2 × 1.0 cm in its greatest dimension which was submitted for histopathological review. The tissue was trisected and submitted entirely in a single cassette for paraffin processing and subsequent histologic evaluation. Three haired skin/subcutaneous tissue sections were examined utilizing H&E stain. Four profiles of vas deferens (epithelial lining confirmed through cytokeratin immunohistochemistry [IHC] staining) which contained abundant luminal spermatozoa were identified within the deep subcutaneous tissue of a single section (Figure 1). Within all three tissue sections were numerous, variably sized nodular accumulations of macrophages (confirmed through CD68 IHC staining) and lymphocytes (confirmed through CD3 IHC staining) surrounding and admixed with cellular debris and extravagated spermatozoa (sperm granulomas). In addition to these nodular areas of inflammation, there were also several elongated tracts of inflammation extending throughout the subcutaneous tissues in two of the three sections. These inflammatory tracts were comprised of thick rim of macrophages and lesser numbers of lymphocytes, plasma cells, and neutrophils surrounding a centralized cavity which often contain proteinaceous fluid, individualized degenerate cells, and/or extravagated spermatozoa (fistulous tracts). One tract was identified to extend to the level of the epidermal–dermal junction. The macrophages lining the fistulous tracts and the macrophages comprising the sperm granulomas were both frequently laden with spermatozoa (Figure 2).

No significant abnormalities were evident on complete blood count and serum chemistry at the time of biopsy other than a mildly elevated creatinine kinase and mild monocytosis. The draining sinus continued to wax and wane over the next several months, and a left inguinal exploration with dissection of the vasocutaneous fistula was subsequently performed. There were numerous adhesions surrounding the vas deferens with very friable tissue. A unilateral left‐sided vasectomy was performed inguinally after repair of the fistula due to the poor tissue quality not allowing for a primary anastomosis or vasovasostomy. After dissecting the vas deferens free from surrounding tissues, it was doubly ligated and divided. The wound was closed in layers with running 4–0 Vicryl suture with non‐overlapping suture lines to minimize fistula recurrence. The monkey had an uneventful post‐operative course and returned to normal behavior after recovery. More than 2 years following surgical repair, the animal is doing well with no evidence of recurrence.

## 4 DISCUSSION

Although complications of seroma, hematoma, urinary retention, hernia recurrence, chronic pain, and surgical site infection in humans have been well described with complications reported in 10% of inguinal hernia repairs,^2^ there is a paucity of data on complications in the NHP inguinal hernia repair population. There has also not been a reported case of a vasocutaneous fistula as a complication of an inguinal hernia repair in either human or non‐human primates.

There is a paucity of data on inguinal hernias reported in rhesus monkeys. Rawlings et al.^6^ published a case report of two rhesus monkeys diagnosed with inguinal hernias in 1971. Krugner‐Higby et al.^7^ reported an increased incidence of inguinal hernias in rhesus monkeys after lead exposure. Contrast herniography and peritoneography have also been reported by James et al.^8^ to assist in the diagnosis of inguinal hernia in NHPs. Berg et al.^9^ reported a rare case of a non‐reducible inguinal hernia containing the uterus and bilateral adnexa in a female rhesus monkey.

To our knowledge, this is the first reported case of a vasocutaneous fistula in a non‐human or human primate. Great care must be taken when performing anatomic hernia repairs to isolate the vas deferens, and the wound should be closed in multiple layers between the vas deferens and the skin. In the rare case of a vasocutaneous fistula, it may be repaired with meticulous surgical technique including gentle tissue handling, minimizing surgical bleeding, and minimal tissue contact techniques.

## 5 CONCLUSIONS

It is possible to form a vasocutaneous fistula in a primate as a complication of inguinal hernia repair. Vasocutaneous fistula surgical repair with closure in multiple layers is the treatment of choice for a vasocutaneous fistula.

## CONFLICT OF INTEREST

None.

## Data Availability

The data that supports the findings of this study are available from the corresponding author upon request.
